# Clinical Features, Diagnosis, Management and Prognosis of Primary Intraocular Lymphoma

**DOI:** 10.3389/fonc.2022.808511

**Published:** 2022-02-03

**Authors:** Xin-yu Zhao, Tian-tian Cheng, Li-hui Meng, Wen-fei Zhang, You-xin Chen

**Affiliations:** ^1^ Department of Ophthalmology, Peking Union Medical College Hospital, Chinese Academy of Medical Sciences, Beijing, China; ^2^ Key Laboratory of Ocular Fundus Diseases, Chinese Academy of Medical Sciences and Peking Union Medical College, Beijing, China

**Keywords:** clinical features, diagnosis, meta-analysis, primary intraocular lymphoma, treatment

## Abstract

**Purpose:**

To evaluate the clinical features, diagnostic techniques, various treatment strategies and prognosis of primary intraocular lymphoma (PIOL).

**Methods:**

The databases PubMed, EMBASE, and Ovid were searched from inception to March 2021 to identify relevant studies. Statistical analyses were performed with R version 3.3.1.

**Results:**

87 studies involving 1484 patients (aged from 14 to 90 years old) were finally included. The pooling results indicated PIOL patients were female, elderly, binocular and B cell type dominated. About 19% have central nervous system (CNS) involvement at the first visit. During follow-up, the incidence of CNS involvement, death rate, 2-year and 5-year survival rate, 1-year and 2-year progression-free survival, and recurrence rate were 58%, 33%, 82%, 70%, 88%, 70%, 44%, respectively. The most common recurrent site was CNS. The delayed diagnosis rate was 85%, the misdiagnosed rate was 64%. The diagnostic technique with the highest positive rate was IL10:IL6>1 of aqueous (98%). The most common symptoms, signs, FFA and OCT features were blurring of vision (72%), vitreous inflammatory opacity (92%), FA/FAF reversal (91%) and hyper-reflective foci in posterior vitreous (53%), respectively. The prognosis of PIOL patients without CNS involvement was obviously better than those with CNS involvement. Overall, intravitreal injection of chemotherapy drug plus systemic chemotherapy (IV+CT) could achieve satisfactory prognosis, the combination of local radiotherapy (RT) could further decrease the recurrent and death rate.

**Conclusion:**

PIOL patients with CNS involvement had significantly worse prognosis. The aqueous humor examination should be regarded as first-line and routine diagnostic technique. IV+CT could achieve satisfactory prognosis, the combination of RT was also beneficial.

## Introduction

Primary intraocular lymphoma (PIOL) is an uncommon form of lymphoma disease, a subtype of primary central nervous system lymphoma (PCNSL) derived from intraocular tissue. PIOL differs from systemic and central nervous system (CNS) lymphoma which metastasize to the eye. It usually originates from vitreoretinal, known as primary vitreoretinal lymphoma (PVRL), and a few derives from uveal and optic nerve (1). PIOL is a life-threatening and blind-causing disease, especially when CNS is involved, resulting in higher mortality and worse prognosis (2). Therefore, accurate pre-CNS-involved diagnosis and effective therapy are of great significance to manage this fatal disease

PIOL usually masquerades as uveitis or other intraocular inflammation due to a wide variety of its manifestation, bringing great confusions to timely diagnosis (3). Some researchers even reported patients with misdiagnosis for more than 2 years (4), putting at risk of the prognosis of PIOL patients. Currently, diagnosis of PIOL mainly relies on the examination of intraocular fluid samples, including cytology, IL10, IL6, IL10/IL6 ratio, flow cytometry and immunoglobulin heavy chain gene rearrangement. The gold standard is still cytology (5, 6). Besides, the specific signs on optical coherence tomography (OCT), fundus fluorescein angiography (FFA) and other fundus imaging techniques also play an important role in the diagnosis and follow-up of PIOL (7, 8).

The treatment to prevent CNS involvement and recurrence is at great debate. The common treatments of PIOL included intravitreal injection (IV) or intrathecal injection (IT) of chemotherapy drug, systemic chemotherapy (CT) and local radiotherapy (RT). Combination therapy may serve as a prospective treatment to prevent or postpone CNS involvement (9–12).

Since delayed diagnosis of PIOL may lead to blindness and life-threatening complication, punctual and accurate diagnosis followed by appropriate treatment are of great significance. Numerous controversies in PIOL still existed. 1) The baseline clinical features of PIOL were reported inconsistently (3, 13–15), like the reported proportion of female cases ranged from 0% to 80% (16, 17), so were the proportion of patients over 60 years old (18, 19); 2) the reported CNS involvement rate at diagnosis varied significantly from 7% to 75.0% (20, 21), and expanded to 20% to 100% in the whole course of disease (22, 23); 3) the positive rate of various diagnostic techniques varied widely, thereby a comprehensive review of previous studies were urgently needed; 4) Many confounding factors, such as CNS involvement and different strategies of management, might affect the prognosis of PIOL, thus more precise evaluations were needed to perfect the treatment strategy. 5) Due to the small sample size of previous studies, the conclusions supported by previous studies are very limited, and their rating of the evidence level were also not high enough to be solid reference.

Until now, few systematic reviews have focused on these controversies of PIOL, a comprehensive and quantitative analysis of current evidence is urgently needed. Thus, our study is conducted to give a full picture of PIOL and evaluate the prognosis of different treatment strategies, providing credible reference for ophthalmologists.

## Methods

This study is based on the standards of “Preferred Reporting Items for Systematic Reviews and Meta-analyses (the ‘PRISMA’ statement)” (24).

### Search Strategy and Study Identification

Human studies in English were searched in PubMed, EMBASE, and Ovid to obtain articles that came out from inception to January 2021, searching by the keywords and their combination: (“Intraocular Lymphoma”[Mesh]) OR ((Primary Vitreoretinal Lymphoma [Title/Abstract]) OR (Primary Intraocular Lymphoma AND ((Humans [Filter]) AND (English [Filter]))) AND ((Humans [Filter]) AND (English [Filter]))). An extensive manual search strategy was also employed for related articles.

### Inclusion Criteria and Exclusion Criteria

Inclusion criteria were: 1) research data on clinical manifestations, diagnosis and treatment of PIOL; 2) At least one of the following outcomes were reported: basic description of the characteristics of PIOL patients, delayed diagnosis rate, misdiagnosed rate, positive rate of various diagnostic techniques, symptoms and signs, features of multi-model imaging, prognosis of different interventions.

Exclusion criteria were: 1) researches about special objects, such as refractory and recurrent PIOL; 2) studies without distinguishing of primary and secondary intraocular lymphoma; 3) review articles; 4) animal experiments or mechanism description; 5) non-English or redundant publications.

### Data Extraction and Assessment of Methodological Quality

Endnote was used to manage references. Two reviewers (X.-y.Z and T.-t.C.) read and screened the title and abstract of articles respectively after removing duplicates. Full texts were browsed for the following valuable information when relevant: first author, year of publication, type of article, cohort size, basic information of patients, diagnostic methods including aqueous humor and vitreous fluid examination, complaints of patients, fundus manifestations obtained by slit lamp, FFA and OCT, treatment strategies and outcomes, development of disease and prognosis, such as CNS involvement, recurrence and death. Authors were contacted for more details when information not available in the literature. A third reviewer (Y.-x.C.) intervened if any disagreement during data acquisition. Notably, the definition of PIOL in some studies was not accurate (no evidence of any other lymphoma at the first relevant visit), so manually identification and elimination was done. Besides, for updated publications with the same cohort of patients of the previous study, the data of the similar case was extracted synthetically and only once.

### Statistical Methods

R version 3.6.3 (R Foundation for Statistical Computing, Vienna, Austria) was used to conduct statistical analysis. Freeman–Tukey variant of arcsine square was used to transform proportions with 95% confidence interval (CI) and statistical heterogeneity between studies was calculated with chi-square test and *I^2^* statistics. For transformed proportion, a fixed-effect model was used if the heterogeneity was low (*P*>0.1, *I^2^* <50%), otherwise sensitivity analysis and subgroup analysis were utilized to find out the source of high heterogeneity (*P*<0.1, *I^2^* >50%), and a random-effect model was used if it could not be eliminated.

Statistical significance was measured with *P*<0.05. Publication bias was verified by funnel plots of the Egger test with statistically significant when *P*<0.05 (25).

## Results

### Study Characteristic

**Figure 1:**
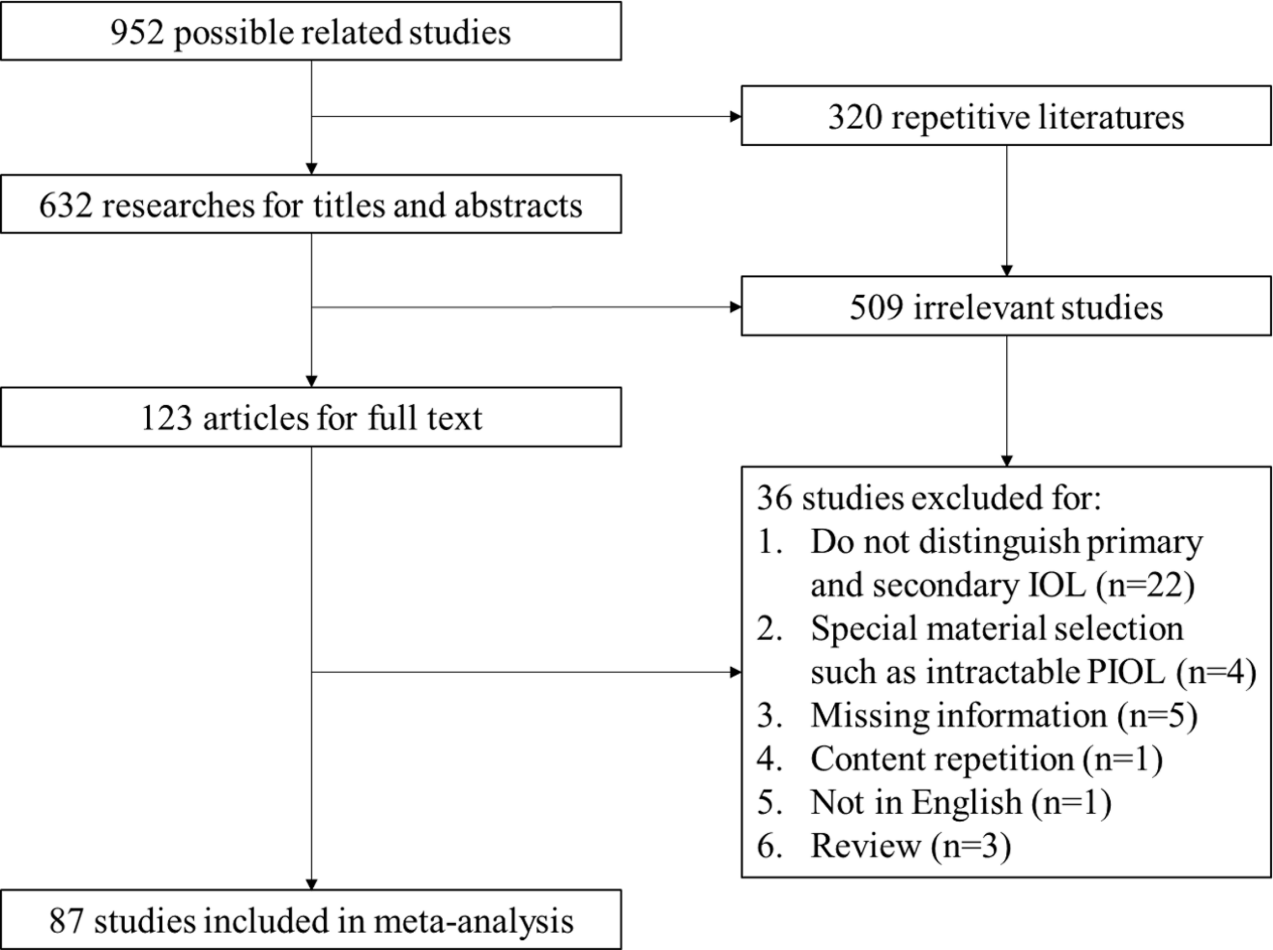
PRISMA flow of screening process. IOL, Intraocular Lymphoma; PIOL, Primary Intraocular Lymphoma.

A total of 952 articles were included as possible related studies. The screening process is shown in **Figure 1**. After removing the repetition and reading abstracts, 123 articles were included for full details, in which 87 articles were finally included. The specific information of these articles is shown in **Table 1**, with 1483 patients in cohort (aged from 14 to 90 years old).

**Table 1:** Main characteristics of the included studies.

First Author	Publication Year	Study Design	Key words	Mean duration of follow-up	Cohort Size (Patients/Eyes)
Zhao, H (7)	2020	Retrospective Study	PVRL, OCT, vMTX	NA	10/18
Park, Y. G (6)	2020	Case Series/Case Report	Aqueous humor, IL-10/6, vMTX	18.35 ± 8.23M	14/23
Arai, A (14)	2020	Retrospective Study	CD79B, Gene expression profiling, PVRL	21-67M	7/10
Zhuang, L (26)	2019	Prospective Cohort Study	Risk, Intraocular involvement, PCNSL	NA	1/NA
Yonese, I (27)	2019	Retrospective Study	Malignant lymphoma, Molecular cytogenetics, PVRL	mean,38M	17/21
Matsuo (28)	2019	Retrospective Case Series	PVRL, CNSL, Vitrectomy cell block	mean, 34M	17/26
Lee, J (29)	2019	Case Series	Immunoglobulin kappa light chain, IL levels, PVRL	4-55M	12/21
Lavine, J. A (30)	2019	Retrospective Case Series	Ultra-widefield&standard FP, Fluorescein angiography, OCT	mean, 33M	23/43
Hah, Y. Y (23)	2019	Retrospective Case Series	IOL, Uveitis, Masquerade syndrome	NA	4/6
Deák, G. G (31)	2019	Retrospective Case Series	OCT, Tomography, PVRL	NA	5/10
de la Fuente, M. I (32)	2019	Clinical Trial	PVRL, Bilateral radiation therapy, PCNSL	mean,68M	12/24
Castellino, A (33)	2019	Retrospective Case Series	VRL, Treatment outcome, Mortality	mean, 36M	33/61
Klimova, A (9)	2018	Comparative Retrospective Study	Prognosis, Treatment medical, PVRL	mean,53M	10/18
Cho, B. J (34)	2018	Comparative Retrospective Study	IOL, Overall survival, VRL	39.4 ± 26.1M	14/23
Barry, R. J (19)	2018	Retrospective Case Series	Early Diagnosis, SD-OCT, PVRL	NA	22/32
Mahajan, S (13)	2017	Case Series/Case Report	PVRL, vMTX, Subretinal deposits	1-64M	11/22
Kuiper, J. J (35)	2017	Case Series	Aqueous humor, Vitreous fluid, PPV	NA	27/NA
Kaburaki, T (10)	2017	Clinical Trial	MTX, PIOL, Radiotherapy	mean, 48.9M	17/27
Saito, T (36)	2016	Case Series	IOL, PVRL, SD-OCT	1-51M	20/26
Milgrom, S. A (11)	2016	Case Series	Radiation therapy, PVRL, Compliance	mean,42M	11/16
Mapelli, C (37)	2016	Retrospective Case Series	PVRL, VRL, Multimodal imaging	NA	6/9
Ma, W. L (12)	2016	Retrospective Case Series	MTX, PIOL, Outcome	mean,40.2M	19/29
Kim, M. M (38)	2016	Case Series	Treatment outcome, Neoplasm recurrence, PIOL	mean,29M	22/38
Keino, H (39)	2016	Retrospective Case Series	IOL, SD-OCT, Diagnosis	mean,20.8M	6/11
Kase, S (40)	2016	Retrospective Observational Case Study	Cytology, Cell block, Masquerade syndrome	NA	6/8
Cimino, L (4)	2016	Retrospective Study	Diagnostic PPV, Subretinal infiltrates, VRL	NA	5/8
Cheah, C. Y (41)	2016	Retrospective Study	MTX, PIOL, Radiotherapy	mean, 50M	11/NA
Akiyama, H (42)	2016	Prospective Study	CNSL, IL-10, IOL	20-75M	18/31
Abu Samra, K (43)	2016	Case Series	Clinical manifestations, IOL, Treatment outcomes	mean,56M	7/13
Riemens, A (44)	2015	Retrospective Cohort Study	Survival rate, Treatment outcome, PIOL	mean, 49M	78/123
Kuiper, J (45)	2015	Comparative Prospective Study	IL, Aqueous humor, PPV	NA	11/NA
Kitiratschky, V. B (46)	2015	Retrospective Observational Case Study	Diagnostic PPV, Intraocular inflammation, VRL	mean, 34M	6/NA
Egawa, M (47)	2015	Retrospective Observational Case Series	Binarization, SD-OCT, PIOL	NA	3/4
Wang, L (48)	2014	Retrospective Study	IL-10, IOL, Single nucleotide polymorphism	6-48M	16/NA
Tuo, J (49)	2014	Prospective Cross-Sectional Study	Diagnosis, MicroRNAs, IOL	NA	17/NA
Teckie, S (50)	2014	Retrospective Study	PIOL, Ocular radiation therapy, PCNSL	mean,25M	18/29
Egawa, M (52)	2014	Retrospective Case Series	SD-OCT, Fundus autofluorescence, PIOL	NA	1/1
Rodriguez, E. F (51)	2014	Retrospectively case series	Vitreous cytology, IOL, OCT	NA	8/NA
Hashida, N (16)	2014	Retrospective Study	PVRL, Prophylactic treatment, CNS involvement	44 ± 18.7M	26/43
Casady, M (8)	2014	Retrospective Case Series	Cytokines, Diagnosis, Fundus autofluorescence	NA	10/18
Hashida, N (16)	2014	prospective study	IVR, PVRL, Treatment outcome	46.6 ± 27.3 M	13/20
Raja, H (53)	2013	Case Report/retrospective case series	IL-10, Aqueous humor, Intravitreal	NA	3/5
Missotten, T (54)	2013	Retrospective Cohort Study	Flow cytometry, Diagnosis, IOL	NA	11/NA
Mikami, R (55)	2013	Retrospective Case Series	Lymphoma, Intraocular, Radiotherapy	mean, 36M	22/38
Fisson, S (56)	2013	Retrospective Study	Diagnosis, IL, PIOL	NA	17/NA
Taoka, K (22)	2012	Case Series	Chemotherapy, Reduced whole-brain radiotherapy, vMTX	mean, 32M	5/8
Kinoshita, Y (57)	2012	Retrospective Case Series	Vitreous fluid cytology, Immunocytochemistry, IOL	NA	8/15
Kimura, K (58)	2012	Retrospective Case Series	IOL, Clinical features, Multicenter study	mean, 41.3M	179/NA
Wang, Y (72)	2011	Retrospective Study	PVRL, Biomarker, PCR	NA	119/NA
Stefanovic, A (59)	2010	Retrospective Study	Therapy, PIOL, Outcome	mean,44M	6/10
Ishida, T (60)	2010	Case Report	Fluorescein angiography, Fundus autofluorescence, OCT	NA	4/5
Sugita, S (61)	2009	Retrospective Study	IL-10, IgH gene rearrangement, Vitreous fluid	NA	13/17
Ohta, K (62)	2009	Retrospective Study	IgH gene rearrangement, IL-10, IOL	7-30M	6/NA
Matsuo, T (63)	2009	Retrospective Study	PIOL, Clonality, vitrectomy cell block	5-45M	7/10
Jahnke, K (64)	2009	Prospective Study	IOL, Ifosfamide, Trofosfamide	mean, 32M	4/NA
Fardeau, C (65)	2009	Comparative Retrospective Interventional Case Series	Diagnosis, Fluorescein angiography, PIOL	NA	53/NA
Wittenberg, L. A (66)	2008	Retrospective Chart Review and Database Study	Cytodiagnosis, Vitreous Body, diagnosis	NA	14/NA
Intzedy, L (67)	2008	Retrospective Case Series	IOL, cytopathology, immunochemistry	NA	7/9
Malumbres, R (68)	2007	Retrospective Study	Immunoglobulin, PIOL, Mutations	NA	5/9
Karma, A (69)	2007	Prospective Noncomparative Study	Diagnosis, Outcome, PIOL	mean, 32M	11/20
Grimm, S. A (70)	2007	Retrospective Chart Review	Ocular lymphoma, PCNSL, Diagnosis	mean, 32M	83/NA
Cassoux, N (5)	2007	Prospective Cohort Study	Aqueous humor, Diagnosis, IL-10	mean, 24M	51/NA
Berenbom, A (71)	2007	Retrospective Interventional Case Series	IOL, Radiotherapy, Chemotherapy	mean, 19M	12/21
Wallace, D. J (72)	2006	Retrospective Case Series	Genetics, Translocation, PIOL	mean,29M	23/NA
Isobe, K (73)	2006	Retrospective Case Series	PIOL, Radiation therapy, Chemotherapy	mean, 19.2M	15/28
Jahnke, K (74)	2005	Prospective Study	Aqueous, Ifosfamide, Trofosfamide	NA	4/NA
Coupland, S. E (75)	2005	Retrospective Study	IgH, CNS neoplasms, IOL	NA	8/NA
Coupland, S. E (76)	2005	Retrospective Study	PIOL, PCR, Sequence analysis	NA	10/NA
Baehring, J. M (77)	2005	Retrospective Study	IOL, IgH gene rearrangement, Chronic vitritis	1-53M	8/NA
Merle-Béral, H (78)	2004	Retrospective Case Series	PIOL, Biological diagnosis, IL-10	NA	36/NA
Lobo, A (79)	2003	Retrospective Study	Diagnostic techniques, Vitreous body, IOL	2-61M	8/NA
Hoffman, P. M (17)	2003	Retrospective Case Series	IOL, Radiation retinopathy, vMTX	14-103M	5/9
Gorochov, G (80)	2003	Prospective Study	Polymorphism, Vitrectomy, PIOL	6-24M	5/8
Coupland, S. E (3)	2003	Retrospective Study	IOL, Chorioretinal biopsy, Immunohistochemistry	mean, 35M	12/19
Chan, C. C (18)	2003	Observational case series	PIOL, Diagnosis, Relay	NA	3/5
Chan, C. C (18)	2003	Retrospective Study	Gene rearrangement, IL-10, IOL	NA	57/NA
Küker, W (81)	2002	Retrospective Study	PCNSL, Uveitis, Ocular manifestations	mean, 6M	4/NA
Shen, D. F (82)	2001	Prospective Study	Microdissection, PIOL, Toxoplasma gondii	NA	10/NA
Akpek, E. K (20)	1999	Prospective Case Series	Diagnosis, IL, PIOL	NA	4/NA
Akpek, E. K (20)	1999	Retrospective Case Series	Intraocular–CNSL, Diagnosis, Treatment outcome	mean,12M	10/18
Chatzistefanou, K (83)	1998	Retrospective Case Series	Uveitis, Pathology, Aged	mean,28M	1/NA
Soussain, C (84)	1996	Prospective Study	IOL, Polychemotherapy, Autologous bone marrow transplantation	13-27M	5/10
Chan, C. C (85)	1995	Prospective Study	IL-10, Diagnosis, Vitrectomy	NA	3/NA
Davis, J. L (86)	1992	Retrospective Case Series	Flow Cytometry, Biomarkers, Diagnosis	NA	4/NA
Strauchen, J. A (87)	1989	Prospective Study	Drug therapy, PIOL, Combined modality therapy	NA	6/9
Siegel, M. J (88)	1989	Case Series	Combined modality therapy, Eye neoplasms, CNS diseases	3-88M	14/24
Klingele, T. G (89)	1975	Retrospective Case Series/Case Report	Ocular reticulum cell sarcoma, Neoplasm metastasis, Diagnosis	NA	5/6

CNS, central nervous system; CNSL, central nervous system lymphoma; IL, interleukin; IgH, immunoglobulin heavy chain; NA, Not available; OCT, optical coherence tomography; PCNSL, primary central nervous system lymphoma; PIOL, primary intraocular lymphoma; PPV, parsplanavitrectomy; PVRL, primary vitreoretinal lymphoma; SD-OCT, spectral domain optical coherence tomography; vMTX, intravitreal injection of methotrexate; VRL, vitreoretinal lymphoma.

### Clinical Features for PIOL

**Figure 2:**
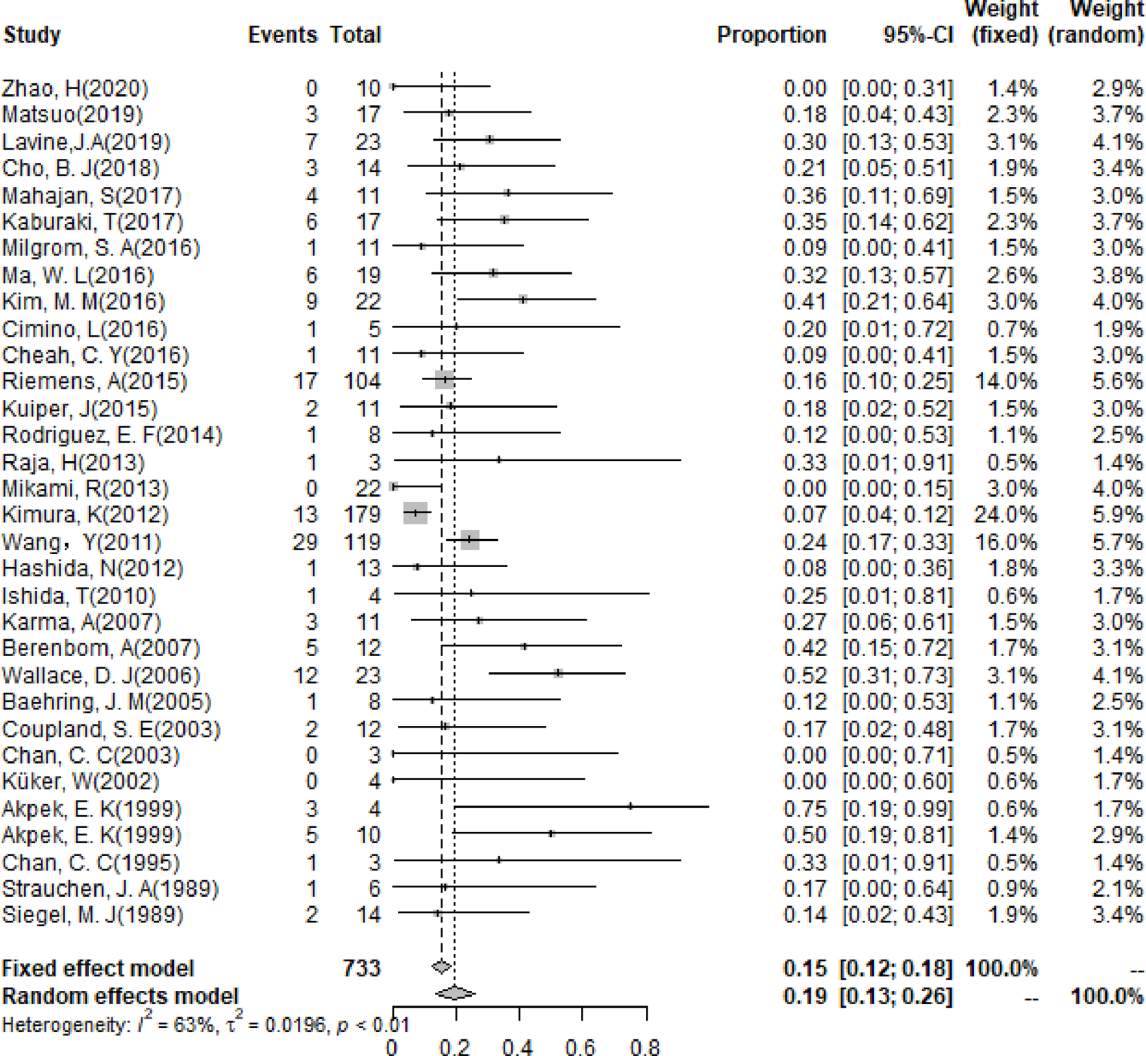
Forest plots of CNS involvement rate at first visit.

**Figure 3:**
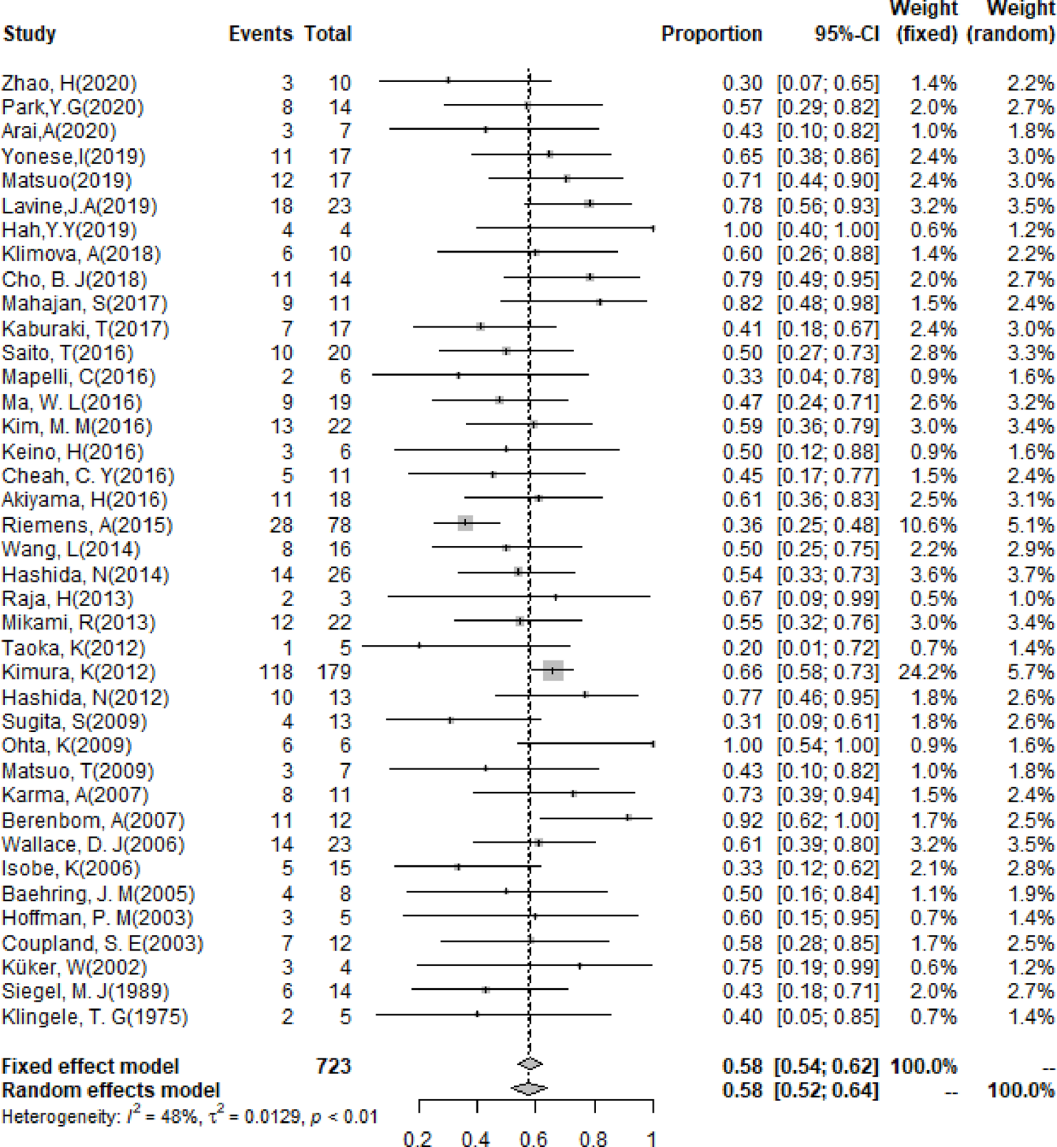
Forest plots of CNS involvement rate during follow-up.

The pooling results of the baseline clinical features of PIOL patients were summarized in **Table 2**. Around 19% (95% CI [13%-26%]) of patients already had CNS involvement when diagnosed (**Figure 2**), which progressed to 58% (95% CI [54%-62%]) during the whole disease course (**Figure 3**), around 33% (95% CI [26%-42%]) of PIOL patients died during follow-up. The most common recurrence site of PIOL was CNS alone (45%, 95% CI [32%-59%]).

**Table 2:** Population distribution and clinical characteristics of primary intraocular lymphoma patients.

Category	No. of studies	Pooled incidence	95% CI		*P* of chi-square	*I* ^2^	Sensitivity analysis	Selected model
**Gender**								
* Male*	69	38%	35%	41%	*P*=0.17	14%	Negative	Fixed-effect model
* Female*	69	62%	59%	65%	*P*=0.15	15	Negative	Fixed-effect model
**Age**								
* <60yrs*	57	26%	22%	30%	*P*<0.01	48%	Negative	Fixed-effect model
* >60yrs*	57	73%	69%	77%	*P*<0.01	43%	Negative	Fixed-effect model
**Ocular involvement**								
* Binoculus*	55	66%	60%	72%	*P*<0.01	0.54	Negative	Random-effect model
* Monocular*	55	34%	28%	40%	*P*<0.01	52%	*Negative*	Random-effect model
**CNS involvement rate at first visit**	46	19%	13%	26%	*P*<0.01	63%	*Negative*	Random-effect model
**Prognosis of PIOL**								
* CNS involvement rate during follow-up*	41	58%	54%	62%	*P*<0.01	48%	Negative	Fixed-effect model
* Death rate during follow-up*	32	33%	26%	42%	*P*<0.01	61%	Negative	Random-effect model
* 2-year survival rate*	18	82%	72%	91%	*P*<0.01	56%	Negative	Random-effect model
* 5-year survival rate*	18	70%	57%	81%	*P*<0.01	66%	Negative	Random-effect model
* 1-year PFS*	5	88%	75%	98%	*P*=0.15	40%	Negative	Random-effect model
* 2-year PFS*	5	70%	44%	91%	*P*=0.07	55%	Negative	Random-effect model
* Recurrence rate*	27	44%	35%	52%	*P*<0.01	64%	Negative	Random-effect model
**Site of recurrence**	4	4%	1%	10%	*P*=0.63	0%	Negative	Fixed-effect model
* CNS alone*	21	45%	32%	59%	*P*<0.01	49%	Negative	Fixed-effect model
* Ocular alone*	21	34%	20%	50%	*P*<0.01	60%	Negative	Random-effect model
* Systemic alone*	21	0%	0%	4%	*P*=0.92	0%	Negative	Fixed-effect model
* CNS and ocular*	21	2%	0%	7%	*P*=0.19	22%	Negative	Fixed-effect model
* Systemic and ocular*	21	0%	0%	0%	*P*=0.94	0%	Negative	Fixed-effect model
**Type of PIOL**								
* B cell*	30	99%	97%	100%	*P*=0.90	0%	Negative	Fixed-effect model
* T/NK cells*	30	1%	0%	2%	*P*=0.97	0%	Negative	Fixed-effect model
**Percentage of PIOL in IOL**	15	48%	42%	54%	*P*=0.65	0%	Negative	Fixed-effect model
**PCNSL with ocular involvement**	17	54%	38%	70%	*P*<0.01	88%	Negative	Random-effect model

CNS, central nervous system; IOL, intraocular lymphoma; PCNSL, primary central nervous system lymphoma; PFS, progression-free survival; PIOL, primary intraocular lymphoma.

### Diagnosis of PIOL

Diagnosis delay of PIOL is defined as the time from onset of ocular symptoms to diagnosis of PIOL. The pooling results indicated that the general delayed diagnosis rate was 85% (95% CI [77% - 93%]), with around 70% (95% CI [53% - 85%]) of PIOL patients having a delayed diagnosis longer than half a year, 37% (95% CI [27% - 48%]) longer than a year. Meanwhile, the misdiagnosed rate was around 64% (95% CI [38% - 70%]). The pooling results of the common symptoms, the positive rate of various diagnostic techniques and the multi-model imaging features were summarized in **Table 3**. The positive rate of aqueous and vitreous examination ranged from 80% to 98%. The most common symptoms, signs, FFA and OCT features were blurring of vision (72%, 95% CI [60% - 83%]), vitreous inflammatory opacity (92%, 95% CI [83% - 99%]), FA/FAF reversal (91%, 95% CI [56% - 100%]) and hyper-reflective foci in posterior vitreous (53%, 95% CI [17% - 88%]), respectively.

**Table 3:** Pooling results about the diagnostic features and techniques of PIOL.

Category	No. of studies	Pooled incidence	95% CI		P of chi-square	I^2^	Sensitivity analysis	Selected model
**Delayed diagnosis rate**	16	85%	77%	93%	*P*<0.01	83%	Negative	Random-effect model
* >0.5y*	11	70%	53%	85%	*P*<0.01	57%	Negative	Random-effect model
* >1y*	11	37%	27%	48%	*P*=0.05	46%	Negative	Fixed-effect model
**Misdiagnosed rate**	10	64%	38%	87%	*P*<0.01	74%	Negative	Random-effect model
**Aqueous humor examination**								
* IL10>50pg/ml*	8	94%	81%	100%	*P*<0.01	66%	Negative	Random-effect model
* IL10:IL6>1*	7	98%	87%	100%	*P*=0.45	0%	Negative	Fixed-effect model
**Vitreous examination**								
* IL10>50pg/ml*	18	88%	82%	94%	*P*=0.32	11%	Negative	Fixed-effect model
* IL10:IL6>1*	24	93%	89%	96%	*P*=0.07	33%	Negative	Fixed-effect model
* Flow cytometry*	8	88%	68%	100%	*P*<0.01	67%	Negative	Random-effect model
* IgH/TCR gene rearrangement*	22	92%	84%	98%	*P*<0.01	75%	Negative	Random-effect model
* Cytological examination*	30	80%	71%	87%	*P*<0.01	69%	Negative	Random-effect model
**Symptoms**								
* Blindness*	3	20%	7%	37%	*P*=0.93	0%	Negative	Fixed-effect model
* Blurring of vision*	18	72%	60%	83%	*P*<0.01	65%	Negative	Random-effect model
* Decreased VA*	12	63%	48%	77%	*P*<0.01	75%	Negative	Random-effect model
* Floater*	19	60%	45%	75%	*P*<0.01	83%	Negative	Random-effect model
* Photopsia*	7	12%	3%	23%	*P*=0.99	0%	Negative	Fixed-effect model
**Signs**								
* Anterior chamber cells*	4	25%	11%	40%	*P*=0.49	0%	Negative	Fixed-effect model
* Aqueous flare*	4	39%	16%	64%	*P*=0.04	64%	Negative	Random-effect model
* Chorioretinal lesions*	4	34%	15%	55%	*P*=0.16	42%	Negative	Fixed-effect model
* Fine KPs*	3	62%	28%	91%	*P*<0.01	81%	Negative	Random-effect model
* Papilloedema of optic nerve*	6	12%	6%	20%	*P*=0.82	0%	Negative	Fixed-effect model
* Retinal detachment*	6	16%	8%	27%	*P*=0.63	0%	Negative	Fixed-effect model
* Retina hemorrhage*	5	9%	3%	16%	*P*=0.79	0%	Negative	Fixed-effect model
* Retinal or subretinal infiltration*	22	61%	47%	74%	*P*<0.01	73%	Negative	Fixed-effect model
* Stellate KPs*	2	8%	2%	18%	*P*=0.55	0%	Negative	Fixed-effect model
* Vitreous inflammatory opacity*	31	92%	83%	99%	*P*<0.01	77%	Negative	Fixed-effect model
**FFA**								
* Diffuse vascular leakage*	3	37%	5%	76%	*P*<0.01	93%	Negative	Random-effect model
* Disk leakage*	3	26%	5%	53%	*P*=0.11	54%	Negative	Random-effect model
* FA/FAF reversal*	3	91%	56%	100%	*P*=0.41	0%	Negative	Fixed-effect model
* Heterogeneous fluorescence*	2	34%	21%	48%	*P*=0.51	0%	Negative	Fixed-effect model
* Hyperfluorescent lesion*	5	20%	8%	36%	*P*<0.01	83%	Negative	Random-effect model
* Round hypofluorescent lesions*	4	37%	5%	75%	*P*<0.01	86%	Negative	Random-effect model
* Window defects*	2	23%	13%	35%	*P*=0.57	0%	Negative	Fixed-effect model
**OCT**								
* Hyper-reflective foci in posterior vitreous*	5	53%	17%	88%	*P*<0.01	94%	Negative	Random-effect model
* Intraretinal lesions*	3	23%	9%	40%	*P*=0.05	67%	Negative	Random-effect model
* IntraRPE lesions*	6	34%	16%	54%	*P*<0.01	77%	Negative	Random-effect model
* Macular edema*	3	12%	6%	19%	*P*=0.35	4%	Negative	Fixed-effect model
* Retinal disorganization*	2	11%	3%	23%	*P*=0.65	0%	Negative	Fixed-effect model
* Retinal hyperreflectivity*	2	45%	28%	63%	*P*=0.52	0%	Negative	Fixed-effect model
* Subretinal lesions*	9	37%	22%	55%	*P*<0.01	75%	Negative	Random-effect model
* SubRPE lesions*	5	23%	7%	45%	*P*<0.01	80%	Negative	Random-effect model

FAF, fundus autofluorescence; FFA, fundus fluorescein angiography; IgH, immunoglobulin heavy chain; KP, keratic precipitates; OCT, optical coherence tomography; RPE, retinal pigment epithelium; TCR, T cell receptor; VA, visual accuracy.

### Subgroup Analysis of Prognosis and Complications of Treatments for PIOL

Subgroup analysis of the prognosis of PIOL was carried out between patients with and without CNS involvement. The detailed results were shown in **Table 4**.

**Table 4:** Pooling results about prognosis of PIOL patients with and without CNS Involvement.

Category	No. of studies	Pooled incidence	95% CI		*P* of chi-square	*I* ^2^	Sensitivity analysis	Selected model
**Death rate during follow-up**								
* CNS involvement*	22	56%	41%	70%	*P*<0.01	60%	Negative	Random-effect model
* Non-CNS involvement*	21	5%	1%	11%	*P*=0.10	32%	Negative	Fixed-effect model
**2-year survival rate**								
* CNS involvement*	11	77%	67%	86%	*P*=0.05	46%	Negative	Fixed-effect model
* Non-CNS involvement*	11	98%	90%	100%	*P*=0.33	13%	Negative	Fixed-effect model
**5-year survival rate**								
* CNS involvement*	11	54%	36%	72%	*P*<0.01	58%	Negative	Random-effect model
* Non-CNS involvement*	11	97%	88%	100%	*P*=0.30	17%	Negative	Fixed-effect model
**Recurrence rate**								
* CNS involvement*	11	70%	51%	87%	*P*<0.01	61%	Negative	Random-effect model
* Non-CNS involvement*	11	20%	11%	31%	*P*=0.72	0%	Negative	Fixed-effect model

The subgroup of various treatments was analyzed, including CT, RT, IV and IT, to figure out their corresponding efficacies and prognosis, especially the involvement of CNS, recurrence rate and mortality. Then, more detailed interventions were evaluated, including CT+RT, CT+RT+IT, IV+CT, IV+CT+RT, IV+IT and IV+ RT, to determine which can give a better prognosis of PIOL (**Table 5**).

**Table 5:** Pooling results about treatment strategy and efficacy for PIOL patients without CNS involvement at first.

Category	CNS Involvement	Recurrence rate	2-year survival rate	5-year survival rate	Death rate during follow-up
CT	100% [75%~100%]	52% [0%~100%]	46% [16%~78%]	50% [11%~89%]	50% [15%~86%]
CT+RT	41% [19%~63%]	36% [19%~55%]	85% [67%~97%]	46% [10%~84%]	25% [9%~44%]
CT+RT+IT	68% [10%~100%]	–	–	–	–
IV	55% [43%~66%]	38% [17%~61%]	90% [64%~100%]	82% [52%~100%]	34% [13%~58%]
IV+CT	43% [27%~59%]	–	100% [91%~100%]	87% [68%~99%]	14% [3%~29%]
IV+CT+IT	–	–	–	–	–
IV+CT+RT	48% [0%~100%]	16% [0%~42%]	–	–	5% [0%~26%]
IV+IT	91% [55%~100%]	–	36% [19%~62%]	–	–
IV+RT	–	–	36% [19%~63%]	–	–
RT	48% [31%~65%]	55% [36%~73%]	98% [81%~100%]	88% [66%~100%]	19% [3%~42%]
**Systemic chemotherapy**					
* With IV*	45% [26%~64%]	65% [29%~95%]	100% [99%~100%]	96% [76%~100%]	4% [0%~20%]
* Without IV*	92% [54%~100%]	34% [2%~75%]	46% [15%~79%]	25% [2%~58%]	52% [27%~76%]
**Systemic chemotherapy**					
* With RT*	46% [21%~72%]	36% [17%~57%]	94% [76%~100%]	55% [21%~87%]	20% [4%~40%]
* Without RT*	92% [54%~100%]	34% [2%~75%]	46% [15%~79%]	25% [2%~58%]	52% [27%~76%]
**Intravitreal injection**					
* With CT*	45% [26%~64%]	65% [29%~95%]	100% [99%~100%]	96% [76%~100%]	4% [0%~20%]
* Without CT*	54% [38%~70%]	40% [14%~68%]	97% [80%~100%]	88% [68%~100%]	27% [5%~54%]

CT, systemic chemotherapy; IT, intrathecal injection of antineoplastic drug; IV, intravitreal injection; RT, local radiotherapy.

### Publication Bias

Using the Egger test (*P* =0.133), no evidence of publication bias was found.

## Discussion

PIOL is an eyesight-damaging and life-threatening disease. Based on currently available research, our study suggested that PIOL patients are female, elderly, binocular and B-cell type dominated. About 19% are CNS-involved when diagnosed. During the follow-up period, the incidence of CNS involvement, death rate, 2-year and 5-year survival rate, 1-year and 2-year PSF, and recurrence rate were 58%, 33%, 82%, 70%, 88%, 70%, 44%, respectively. The most common recurrent site was CNS. About half of IOL was PIOL, as well as PCNSL with ocular involvement. The delayed diagnosis rate of PIOL was extremely high, so was the misdiagnosed rate. IL10:IL6>1 of the aqueous had the highest positive rate among laboratory examinations (98%, 95% CI [87% - 100%]). The most common symptoms, signs, FFA and OCT features were blurring of vision, vitreous inflammatory opacity, FA/FAF reversal and hyper-reflective foci in posterior vitreous, respectively. Overall, the prognosis of PIOL patients without CNS involvement was much better than those with CNS involvement, such as death rate (5% versus 56%), 2-year survival rate (98% versus 77%), 5-year survival rate (97% versus 54%) and recurrence rate (20% versus 70%). IV+CT was recommended as a satisfactory treatment strategy with less burden and side effects, the combination of RT might further benefit in decreasing the recurrent and death rate.

### Clinical Features of PIOL

The basic clinical characteristics about PIOL were quite controversial, and the limited sample size and inconsistent data of previous studies could not offer us a reliable impression. For example, previous studies demonstrated a large variation in the proportion of female in PIOL patients (20% to 100%) (16, 17), binocular involvement (22% to 100%) (32, 67), and CNS involvement rate when diagnosed (7% to 75%) (20, 21). By pooling the data of all currently available studies, our study gave a solid reference when explaining the clinical features of PIOL to patients.

Generally, PIOL began as monocular onset, and more than half progressed to binocular, for the destruction of blood-eye barrier might stimulate the fellow eye. It occurred mainly in the elderly due to weak immune system and mutation accumulation (1). About 19% of PIOL patients are already CNS-involved when diagnosed, defined as the ocular symptoms occurred before the diagnosis of CNS involvement, which further progressed to 58% during the whole disease course. When giving a PIOL diagnosis, we must clarify whether CNS were involved, as their prognosis was very different. Thus, routine head magnetic resonance imaging (MRI) and even cerebrospinal fluid (CSF) examination should be conducted at baseline.

Most PIOL were reported to be B cell types (99%, 95% CI [97% - 100%]). Two hypotheses were proposed to explain its origin. Chan et al. (18) detected B-cell receptors (CXCR4 and CXCR5) on the surface of lymphoma cells, and found the corresponding ligand B-lymphocytic chemoattractant (BLC) on RPE, indicating the abnormal expression of BLC on lymphoma cells are attracted to eye tissues through chemotaxis. Shen et al. (82) found that infections such as HHV8 virus and Toxoplasma may be associated with some PIOL, triggering B cell monoclonal proliferation and thereby lymphoma. T cell-derived PIOL was very rare, which mainly involves vitreoretinal. Its population distribution and ocular manifestations were generally similar to B cells types, which could only be differentiated by laboratory examinations (90).

### Signs and Imaging Findings

Our study suggested that the leading three complaints of PIOL patients were blurred vision, decreased vision acuity and floater, the most common signs were vitreous opacity, fine KPS and retinal or subretinal infiltration. These were mainly caused by the aggressive destruction of the retinal photosensitive structure by lymphoma cells’ invasion or the production of space-occupying turbidity in the vitreous. Besides, PIOL could not only be manifested as fine KPs, but also as stellate KPs.

In OCT, more than half of the patients had hyper-reflective foci in posterior vitreous, combined with retinal hyperreflectivity, subretinal lesions and intra-RPE lesions, representing the functional abnormality and structural interruption caused by the infiltration of different degrees of lymphoma cells in different layers of the retina, which would not appear in eye inflammatory diseases (7). OCT could show more detailed features of lymphoma infiltration and have definite diagnostic significance for PIOL, especially when dense vitritis occurred or the lesions were small. Zhao et al. also suggested that OCT could be used as a non-invasive method to reflect the therapeutic effect and progress of PIOL (7).

The most frequent finding of FFA was FA/FAF reverse (91%, 95% CI [56% - 100%]), defining as high autofluorescence spot on FAF corresponding to a low autofluorescence spot without leakage in this region on FFA. The incidence of other signs was relatively lower than 50%, such as diffuse vascular leakage corresponding to lesion and surrounding small blood vessels, which might be an earlier sign of PIOL before the formation of subretinal lesions.

### Laboratory Examination and Diagnosis

The examination of aqueous humor and vitreous fluid was of great significance for the diagnosis of PIOL. However, the positive rate of each index reported in previous studies fluctuated greatly, making it difficult to launch parallel comparison (5, 6, 16). Our study evaluated and reported the utility of different laboratory examinations, providing reference for diagnosis of PIOL.

Pars plana vitrectomy (PPV) was the last resort for the diagnosis of PIOL with a reliable positive rate due to its damaging operation. Our study indicated that the positive rate of gene rearrangement of IgH and T cell receptor (TCR) was 92% (95% CI [84%-98%]). For cytokine test, positive rate of IL10>50pg/ml (29) was 88% (95% CI [82%-94%]), and the sensitive of IL10/IL6>1 was higher as 93% (95% CI [89%-96%]). Besides, flow cytometry could be used to detect markers on cell surface if samples were enough, with the positive rate of 88% (95% CI [68%-100%]). In addition, observing lymphoma cells in cytological examination directly was still the gold standard with the highest specificity, but the sensitivity was only around 80% (95% CI [71%-87%]), resulting from the fragility of tumor cells with the degeneration caused by untimely inspection. Resultantly, multiple inspections or other markers were needed.

Aqueous humor tests might be an ideal technique due to minimal trauma and valid positive rate compared to vitreous fluid. The most promising one was IL10/IL6>1 with a positive rate near 98% (95% CI [87%-100%]) in PIOL patients. The positive rate of IL10>50pg/ml (5) was also as high as 94% (95% CI [81%-100%]). IL10 was expressed by malignant tumor cells, inhibiting various immune-related cell populations to achieve immune escape, while the rise of IL6 occurred in inflammation indicated stronger immune response. Thus, the levels of IL10 and IL6 have a great potential to distinguish PIOL from ocular inflammation to recognize camouflage syndrome.

According to clinical expression, the positive rate of vitreous fluid examination should be the highest. Interestingly, our analysis showed that of aqueous humor tests was higher. The possible reasons could be as follows: 1) The collection of aqueous humor was so convenient that most operation errors and detection delay were avoided; 2) Cytokines level aqueous humor and vitreous fluid might be generally equivalent because cytokines produced by tumor cells in vitreous cavity could smoothly diffuse into aqueous humor with an ideal concentration; 3) The absence of microenvironment effect of tumor cells reduced the difficulty of cytokines detection in aqueous humor. Meanwhile, the possible reasons for the relatively low positive rate of diagnostic vitrectomy specimens included difficulty of sample acquisition, inspection delay, and sample dilution et al. Besides, multiple PPV may increase the risk of lymphoma spread through the sclerotomy port to the epibulbar space (23). Therefore, we suggest that for patients with clinical manifestations and imaging characteristics supporting PIOL, the aqueous humor test should be the first choice for confirming the diagnosis, diagnostic PPV should be the last resort.

For accurate diagnosis, when a patient experience vision loss, fine KPs, retinal or subretinal infiltration and vitreous inflammatory opacity, hyper-reflective foci in posterior vitreous, retinal hyperreflectivity on OCT, FA/FAF reversal on FFA, ophthalmologists should consider the possibility of PIOL, especially in the elderly. Aqueous humor test and cranial MRI should be routinely conducted to prompt the need for further invasive procedures to make timely diagnosis and treatment.

### Treatment and Prognosis

Subgroup analysis indicated that the prognosis of PIOL patients without CNS involvement was much better than those with CNS involvement, such as death rate during follow-up (5% versus 56%), 2-year survival rate (98% versus 77%), 5-year survival rate (97% versus 54%) and recurrence rate (20% versus 70%). Thus, clarification of CNS involvement is of great significance for the prognosis to PIOL patients.

As for the treatment of PIOL, many controversies still existed due to small sample size, and thereby expanding the size of study is the key. Therefore, our study integrated all the available data to provide a solid reference for the management of PIOL.

According to **Table 5**, IV+CT could achieve a satisfactory prognosis regarding CNS involvement, 2-year and 5-year survival rate, and death rate during follow-up. IV+CT+RT could achieve the lowest recurrent rate, while survival and death rate during follow-up could not be evaluated due to limited data. For CT+RT, the CNS involvement rate was the lowest, while the 2-year and 5-year survival rates were not satisfactory. Subgroup analysis indicated that the prognosis of CT combined with IV was significantly better than that of CT alone considering CNS involvement (45% versus 92%), death rate (4% versus 52%), 2-year survival rate (100% versus 46%) and 5-year rate (96% versus 25%). Similarly, CT combined with RT could improve prognosis more than CT alone. Therefore, we believe that the current evidence supports CT+IV be used as the first-line treatment for the PIOL patients. If possible, the combination of RT could further decrease the recurrence rate and death rate (14% versus 5%) during follow-up. Other more aggressive treatment showed limited efficacy in studies and were not recommended.

### Strengths and Limitations

To the best of our knowledge, this is the first meta-analysis evaluating all available evidence of PIOL from different angles. This study characterized with the largest sample size and the highest level of evidence, which may provide solid references for ophthalmologists, contribute to a better understanding of the disease course and facilitate smoother communication with patients.

However, some limitations still existed. Firstly, the included studies span a wide period time from 1975 to 2020. Although the heterogeneity and publication bias were properly controlled, a difference existed in baseline features of patients (institutional referral bias) and medical techniques, might influence the results of our study. Besides, due to the lack of data in IV+CT+IT or IV+CT+RT, the efficacy of some strategies could not be comprehensively evaluated. To evaluate the prognosis of different treatments, we only focused on major treatment strategy, giving an overall direction and propose that IV + CT could achieve satisfactory prognosis. More large-scale clinical trials were needed to further explore the specific dose and refinement method in the future. We really hope our study could service as directional reference for future clinical trials and research. Secondly, genetic features of PIOL were of great value for the diagnosis and prognosis. Yonese et al. (27) detected that CD79B(Y196) in vitreous DNA might contribute to the confirmation of the diagnosis, and Arai et al. (14) regarded that CD79B mutations showed potential to serve as prognostic markers for CNS progression. Another study conducted by Wallace et al. (72) described that the bcl-2 t(14,18) translocations, the bcl-10 gene, and expression of bcl-6 mRNA in PIOL when compared with other systemic lymphomas, providing useful adjuncts to the pathologic diagnosis of this complex disease. But the number of articles were far too small for conducting a reliable meta-analysis, we did not report them in our study. Genetic features were indeed a potential diagnostic and prognostic marker for PIOL. Thus, we should pay more attention on it in future study. Thirdly, the visual outcome was also of great importance for the surviving PIOL patients. While the current data of visual outcomes was too limited for conducting a reliable meta-analysis, so we did not report it in our study. Thus, we suggest that further study should not only focus on the survival of the PIOL patients, but also pay more attention to the visual outcomes.

## Conclusion

PIOL is an eyesight-damaging and life-threatening disease, patients with CNS involvement had a significantly worse prognosis. The aqueous humor examination should be regarded as a first-line and routine diagnostic technique. IV+CT could achieve a satisfactory prognosis with less burden and side effects, the combination of RT could further decrease the recurrent and death rate during follow-up.

## Data Availability Statement

The original contributions presented in the study are included in the article/supplementary material. Further inquiries can be directed to the corresponding author.

## Author Contributions

X-yZ designed this subject, carried out statistical analysis and revised the manuscript. T-tC conducted literature searching and data extraction, then drafted manuscripts. L-hM and W-fZ helped revised the manuscript. Y-xC coordinated and participated in the entire process of composing the manuscript. All authors read and approved the final manuscript.

## Conflict of Interest

The authors declare that the research was conducted in the absence of any commercial or financial relationships that could be construed as a potential conflict of interest.

## Publisher’s Note

All claims expressed in this article are solely those of the authors and do not necessarily represent those of their affiliated organizations, or those of the publisher, the editors and the reviewers. Any product that may be evaluated in this article, or claim that may be made by its manufacturer, is not guaranteed or endorsed by the publisher.
